# Glutamate metabotropic receptor 4 in breast cancer: a potential and specific target for chimeric antigen receptor therapy

**DOI:** 10.3389/fonc.2025.1654977

**Published:** 2026-01-07

**Authors:** Yien Xu, Ayidana Hayierhan, Zexiao Chen, Yumei Ma, Wenjun Zhang, Chaoliang Xu, Ruyi Mei, Xiaoling Zhou, Yuhao Zhu, Pingnan Sun, Jundong Wu

**Affiliations:** 1 The Breast Center, Cancer Hospital of Shantou University Medical College, Shantou, Guangdong, China; 2 Department of Radiation Oncology, Shantou Central Hospital, Shantou, Guangdong, China; 3 Stem Cell Research Center, Shantou University Medical College, Shantou, Guangdong, China; 4 Shantou University Medical College, Shantou, Guangdong, China; 5 Department of Gynecology of the First Affiliated Hospital, Shantou University Medical College, Shantou, Guangdong, China

**Keywords:** breast cancer, car-t, GRM4, solid tumor, tumor-specific antigen

## Abstract

**Background:**

Despite the revolutionary success of chimeric antigen receptor (CAR) therapy in hematologic malignancies, its application in solid tumors is hindered by the scarcity of tumor-specific membrane antigens rigorously validated in clinical specimens. Here, we identified glutamate metabotropic receptor 4 (GRM4) as a novel target with dual advantages: breast cancer (BC)-predominant membrane expression and restricted normal tissue distribution, potentially circumventing on-target off-tumor toxicity.

**Methods:**

Through integrative multi-database analysis (DESeq2/edgeR/limma differential screening, CellMarker filtration, the Human Protein Atlas database validation), GRM4 was prioritized. Its expression was validated in non-malignant organs [immunohistochemistry (IHC)], BC cell lines [western blot (WB)/quantitative polymerase chain reaction (qPCR)/immunofluorescence (IF)], 158 BC clinical samples with paired para-cancerous tissues (IHC). Subcellular localization, tumor proportion score, and subtype-specific distribution were analyzed. Clinical correlations and survival outcomes were evaluated using chi-square tests and Kaplan-Meier analysis.

**Results:**

Membrane expression was confirmed by IHC in 35.44% of clinical cases, and its presence in breast cancer cell lines was validated by WB, qPCR, and IF. GRM4 exhibited tumor-specific membrane/cytoplasmic expression in 80.38% of BC patients (127/158) across all subtypes (≥70% positivity), with 51.27% showing >50% tumor cell positivity. Critically, GRM4 was absent in normal breast/para-cancerous tissues and confined to the brain in non-malignant organs. While GRM4 correlated with advanced clinical stage (p=0.025) and age (p=0.026), it was independent of overall survival (p=0.449).

**Conclusions:**

GRM4 emerges as a novel CAR-associated target for breast cancer, demonstrating tumor-specific overexpression, brain-restricted normal expression, and pan-subtype applicability with potential on-target off-tumor effect.

## Background

Female breast cancer (BC) is the most common cancer among women in terms of both incidence and mortality, and was the second leading cause of global cancer incidence in 2022 (1). According to the International Agency for Research on Cancer (IARC), with about 2.3 million new cases annually, breast cancer has become the leading cause of cancer-related deaths globally (1). Current treatment strategies for breast cancer typically involve a multidisciplinary approach, including surgery, radiotherapy, chemotherapy, and endocrine therapy (2) (^文献^:Breast Cancer: An Overview of Current Therapeutic Strategies,Challenge,and Perspectives). However, effectively controlling the progression of advanced-stage breast cancer remains a challenge. Thus, exploring novel treatment modalities has been a focus of research in recent years.

In recent years, chimeric antigen receptor T-cell (CAR-T) therapy has achieved remarkable success in treating hematological malignancies, leading to significant and durable responses in patients with acute lymphoblastic leukemia (ALL) (3), lymphoma (4–7), and multiple myeloma (8). In breast cancer treatment, CAR-T therapy shows promise. Currently, several targets, such as Her-2 (NCT04650451), ROR1 (NCT02706392), and mesothelin (NCT02580747), are being studied in clinical trials of CAR-T cell therapy for breast cancer. Nevertheless, clinically applicable targets are still rare, and further exploration is needed.

Notably, the on-target off-tumor effect, a principal side effect of CAR-T therapy, requires special attention. This is because some targets are expressed in the normal tissues and organs and may be attacked by CAR-T cells (9). Clinical studies using CAR-T cells targeting antigens shared by malignant and non-malignant tissues have reported varying degrees of on-target off-tumor effects (10–12).

Currently, tumor biomarkers mainly include tumor-associated antigens (TAAs) such as Her-2 (13) and CD19 (14). Targeting TAAs has a potential risk of on-target off-tumor side effects, as CAR-T cells designed to recognize these antigens may also attack normal tissues (15, 16). Andrew J. Hou (17) noted that addressing the heterogeneity of tumor antigens is crucial to preventing misidentification and destruction by CAR-T cells. Therefore, screening for relatively specific targets is essential for the safety and efficacy in treating solid tumors with CAR-T cell therapy.

Recently, proteins with ectopic expression, such as CENP-A (18) and COL5A2 (19), have been the focus of research, but few have been clinically applied. Many researchers use bioinformatics analysis for differential gene analysis to select specific biomarkers. Although the goal is to identify tumor-specific targets, it is difficult to find a marker expressed only in tumors. Thus, the focus is on tumor-associated antigens. Our group has previously investigated several tumor-associated target in cancer, such as CD22 in breast cancer (20) and esophageal cancer (21), CD276 in esophageal cancer (22). Some targets of interest are ectopically expressed in human organs and sourced from the CellMarker database (23), an updated database of manually curated cell markers, in humans, based on single-cell RNA-sequencing (scRNA-seq). Here, we used these methods first to identify GRM4 as a potential CAR-associated target with a low risk of causing severe side effects.

## Materials and methods

### Bioinformatics analysis

On the RStudio platform, differential expression analysis between breast cancer and normal samples was conducted using the DESeq2, edgeR, and limma packages. The study incorporated 113 normal breast tissues and 1104 tumor tissue samples from The Cancer Genome Atlas (TCGA) database (https://portal.gdc.cancer.gov/projects/TCGABRCA). The differential expression criteria were set as log fold change (logFC)≥4 and p ≤0.05.

Differentially co-expressed genes were intersected with the CellMarker database (23) to identify tissue-specific genes. Then, their subcellular localization was analyzed using the Human Protein Atlas available from http://www.proteinatlas.org (24), classifying them into intracellular, secreted, or membrane-localized proteins. Their subcellular localization was analyzed using the Human Protein Atlas database (24) (**Additional File 4**).

Moreover, the expression of GRM4 in 291 adjacent normal tissues and 1085 tumor tissues from the Gene Expression Profiling Interactive Analysis database (GEPIA) was analyzed to evaluate its differential expression (25). For membrane-localized genes, their expression profiles across normal organs from Human Protein Atlas (http://www.proteinatlas.org) (26) were further analyzed to identify potential breast cancer therapeutic targets.

### Tumor tissue from patients with breast cancer

A comprehensive dataset consisting of 158 breast cancer tissue samples was collected from patients who underwent radical resection for breast cancer at the Cancer Hospital of Shantou University Medical College between 2015 and 2018. Normal organ slides were also procured from the same institution. Diagnostic criteria for these patients were according to the guidelines specified in the 8th edition of the American Joint Committee on Cancer (AJCC) staging system (27) for breast cancer. Among them, 22 breast cancer patients were at stage III, characterized by deeper tumor invasion and more lymph node metastasis, while 136 were at stage I–II. Most patients in our study are at early stage. In this study, only patients who had not received chemotherapy or radiotherapy before surgery were included, as these treatments might potentially induce changes in the expression of target genes. The ages of all patients ranged from 28 to 86. Ethics approval for human subjects was approved by the Ethics Committee of Shantou University Medical College (No. 2024068), and informed consent was obtained from each patient. Clinical trial number: not applicable. All procedures performed in studies were in accordance with the 1964 Helsinki declaration and its later amendments or comparable ethical standards.

### Cell culture

The 293T, MCF7, and MDA-MB-231 cell lines, and mesenchymal stem cells (MSCs) (negative control) were cultured in high-glucose DMEM medium (SH30243.01, HyClone, Cytiva, South Logan, UT, USA) supplemented with 10% fetal bovine serum (10099141C, Gibco, Australia) and antibiotics (Pen/Strep, 15140-122, Gibco, Waltham, MA, USA). For passaging, cells were digested with 0.05% trypsin-EDTA (25200072, Thermo Fisher Scientific, Waltham, MA, USA). 293T cells were positive controls for GRM4, while MSCs were negative controls.

### RNA extraction and quantitative polymerase chain reaction

Total RNA was extracted from breast cancer cell lines (MCF7 and MDA-MB-231) using RNAiso (RNAiso Plus, 9109, Takara). Subsequently, cDNA synthesis was performed with a ReverTra Ace qPCR RT Kit (FSQ-101, TOYOBO). To quantitatively evaluate gene expression, qPCR was carried out using SYBR Green Master Mix (A25742, Thermo Fisher Scientific). Expression levels of target genes were normalized to the housekeeping gene GAPDH. Finally, the RT-qPCR data were analyzed by the 2^-ΔΔCT method. Primer sequences used are presented in **Additional file 1**.

### Western blot analysis

Western blot analysis was conducted on MCF7 and MDA-MB-231 breast cancer cell lines, and 293T (positive control) cells. Protein concentrations were quantitatively assessed using a BCA assay (P0010, Beyotime, Shanghai, China). Subsequently, the lysates were fractionated via 7.5% (w/v) SDS-PAGE gel electrophoresis and transferred onto polyvinylidene difluoride (PVDF) membranes. The membranes were then incubated with primary anti-GRM4 antibody (ab53088, Abcam) at a dilution of 1:1000, followed by an HRP-conjugated anti-rabbit secondary antibody (BOSTER, BA1054). The resulting signal was visualized and analyzed using an Odyssey Imaging System (ChemiDoc XRS+ system, Bio-Rad, Hercules, CA, USA).

### Immunofluorescence

Cell lines were fixed with cold methanol for 15 min, washed with PBST (PBS, ZLI-9062, ZSGB-BIO, Beijing, China; TWEEN 20, V900548, Sigma-Aldrich, Darmstadt, Germany), incubated with primary anti-GRM4 antibody (1:100, ab53088, Abcam, Cambridge, UK) overnight at 4°C, and visualized using appropriate secondary fluorescent antibodies (Alexa Fluor^®^488, ab53088, Abcam). Cells were subsequently counterstained with DAPI (diluted with water at a ratio of 3:7, C1006, Beyotime, Shanghai, China) and images were acquired from a fluorescence microscope (Axio Observer A1, Zeiss, Oberkochen, Germany).

### Immunohistochemical staining of breast cancer specimens

Immunohistochemistry (IHC) was performed on 158 formalin-fixed and paraffin-embedded breast cancer specimens. Four-micrometer-thick sections of breast cancer tissue and cerebellum (positive control for GRM4) underwent antigen retrieval in a pressure cooker for 8 minutes. Brain sections incubated with GRM4 antibody were used as positive controls, whereas those incubated with phosphate-buffered saline (PBS) were negative controls. Nonspecific binding sites were blocked using normal serum (ZLI-9024, ZSGB - BIO, Beijing, China). After the blocking step, sections were incubated with a primary anti-glutamate metabotropic receptor 4/GRM4 (1:200, ab53088, Abcam) at 4°C for 14 hours. Subsequently, a two-step detection system with a secondary antibody (PV-9000, ZSGB-BIO, China) was utilized. DAB (DAB, ZLI-9018, ZSGB-BIO, Beijing, China) was used for color development, and slides were then counterstained with hematoxylin (3201111, Wexis, Guangzhou, China). Our IHC findings were categorized by H-scores, where the staining intensity was defined as follows: 0 denoted an absent expression of the target antigen within tumor cells, 1 signified weak positive expression, 2 corresponded to moderate positivity, and 3 represented a high degree of positive expression. Furthermore, the proportion of positive tumor cells was categorized as: 0 encompassing a range from 0% to 10% of positive cells, 1 spanning from 10% to 25%, 2 extending from 26% to 50%, 3 covering 51% to 75%, and 4 indicating a proportion ranging from 75% to 100% of positive tumor cells. The H-score, a pivotal metric in this analysis, was derived by the summation of the staining intensity score and the percentage of positive cells.

### Statistical analysis

For statistical analysis, SPSS software (Version 20, IBM, Armonk, NY, USA) was employed. The chi-square and Fisher’s exact tests were used to analyze the relationship between clinical pathological features and IHC staining. A p-value less than 0.05 was considered statistically significant. Additionally, to assess the correlation between the overall survival of breast cancer patients and GRM4 expression levels in breast cancer specimens, SPSS software was applied.

## Results

### High expression of GRM4 in breast tumors was revealed from bioinformatics analysis

**Figure 1:**
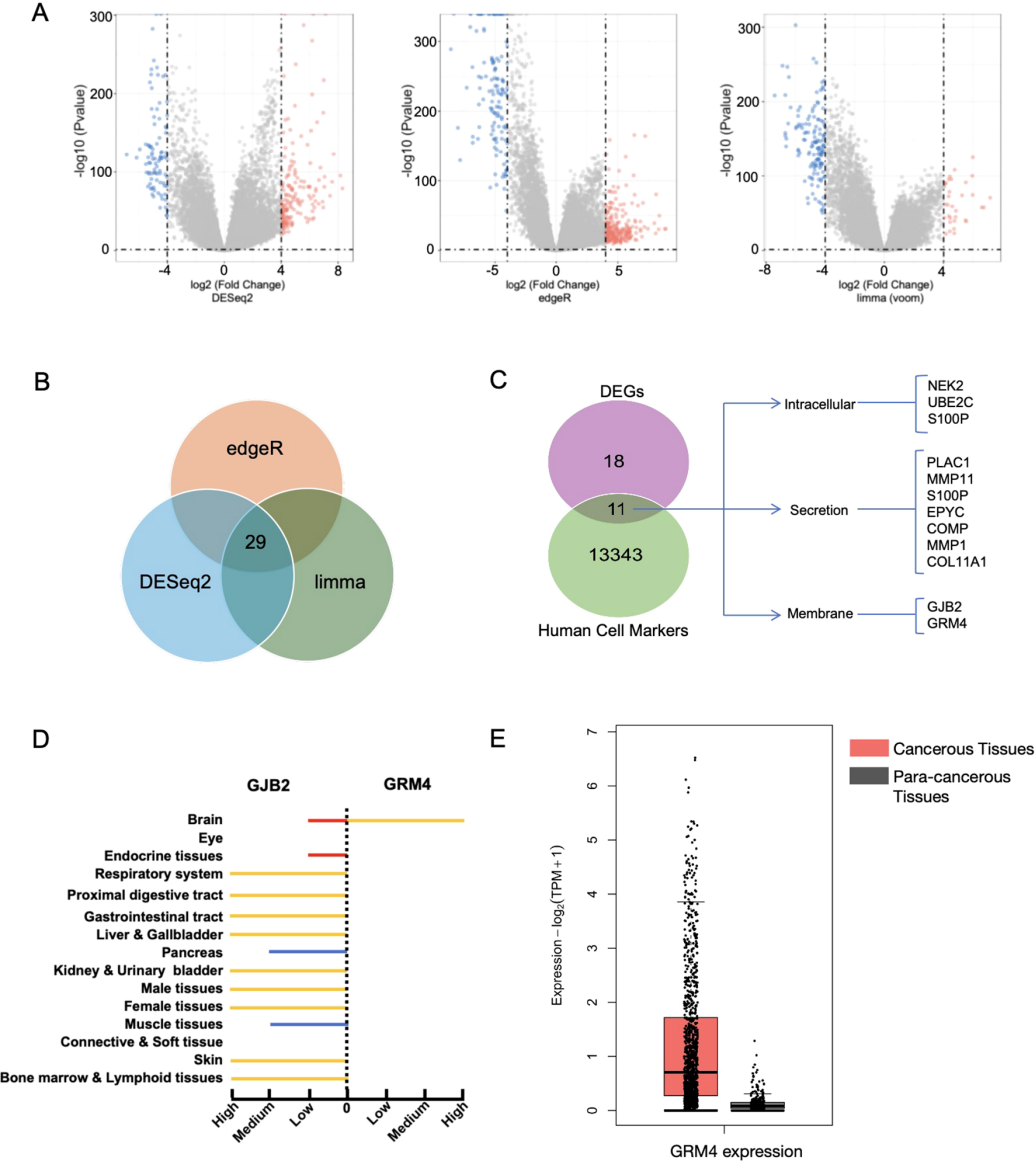
Differential expression analysis and tissue-specific gene identification in breast cancer. Bioinformatic screening identified GRM4 as a candidate target. **(A)** Volcano plots showing differentially-expressed genes between breast cancer and normal samples, using DESeq2 (left), edgeR (middle), and limma (right) packages. Dashed lines indicate the threshold for differential expression (logFC ≥ 4, p ≤ 0.05). **(B)** Venn diagram illustrating the intersection of up-regulated DEGs identified by DESeq2, edgeR, and limma packages. Twenty-nine genes were selected. **(C)** Venn diagram shows that among 29 genes selected by the three packages, 11 tissue-specific genes were found. Data was obtained from the Human Cell Markers database (http://117.50.127.228/CellMarker/index.html). Gene subcellular localization: intracellular (pink), secreted (green), and membrane (blue). **(D)** Protein expression scores from the HPA for GJB2 and GRM4 across various human organs. Expression levels are categorized as low (red), medium (blue), and high (yellow). Data were obtained from Human Protein Atlas Dataset available from proteinatlas.org. (https://www.proteinatlas.org/). **(E)** Box plot showing that GRM4 is higher in tumor tissues (N = 1085) compared to normal tissues (N = 291) from the GEPIA database (http://gepia.cancer-pku.cn/index.html).

To select some specific marker expressing in breast cancer, we adapt bioinformatics analysis. Using differential expression analysis (logFC ≥ 4, p-value ≤ 0.05), we identified 170, 242, and 33 upregulated genes using DESeq2, edgeR, and limma packages, respectively (**Figure 1A**, **Additional File 2**). The intersection of these results yielded 29 commonly upregulated genes (**Figure 1B**). Intersecting these 29 genes with the CellMarker database resulted in 11 tissue-specific genes (**Figure 1C**) (23). Subcellular localization analysis revealed that 3 genes were predominantly expressed intracellularly, 7 were secreted proteins, and 2 were localized to the cell membrane. The intracellular proteins included NEK2, UBE2C and S100P. The secreted proteins included PLAC1, MMP11, S100P, EPYC, COMP, MMP1 and COL11A1. The membrane proteins included GJB2 and GRM4 (**Figures 1C, D**).

Further analysis of these two membrane-localized genes showed that GRM4 was exclusively expressed in the brain and undetectable in other normal tissues, whereas GJB2 was highly expressed in multiple normal tissues (**Figure 1D**).

Additionally, GEPIA database analysis revealed that GRM4 was expressed at low levels in 291 adjacent normal tissues but was highly expressed in 1085 tumor tissues, with a significantly higher expression in tumor tissues compared to adjacent normal tissues (**Figure 1E**). Due to the existence of the blood-brain barrier, CAR-T cells do not easily traverse it (28). Thus, GRM4 was preliminarily identified as a potential CAR-T therapy target for breast cancer.

### GRM4 expression is confined to the brain in normal tissues

**Figure 2:**
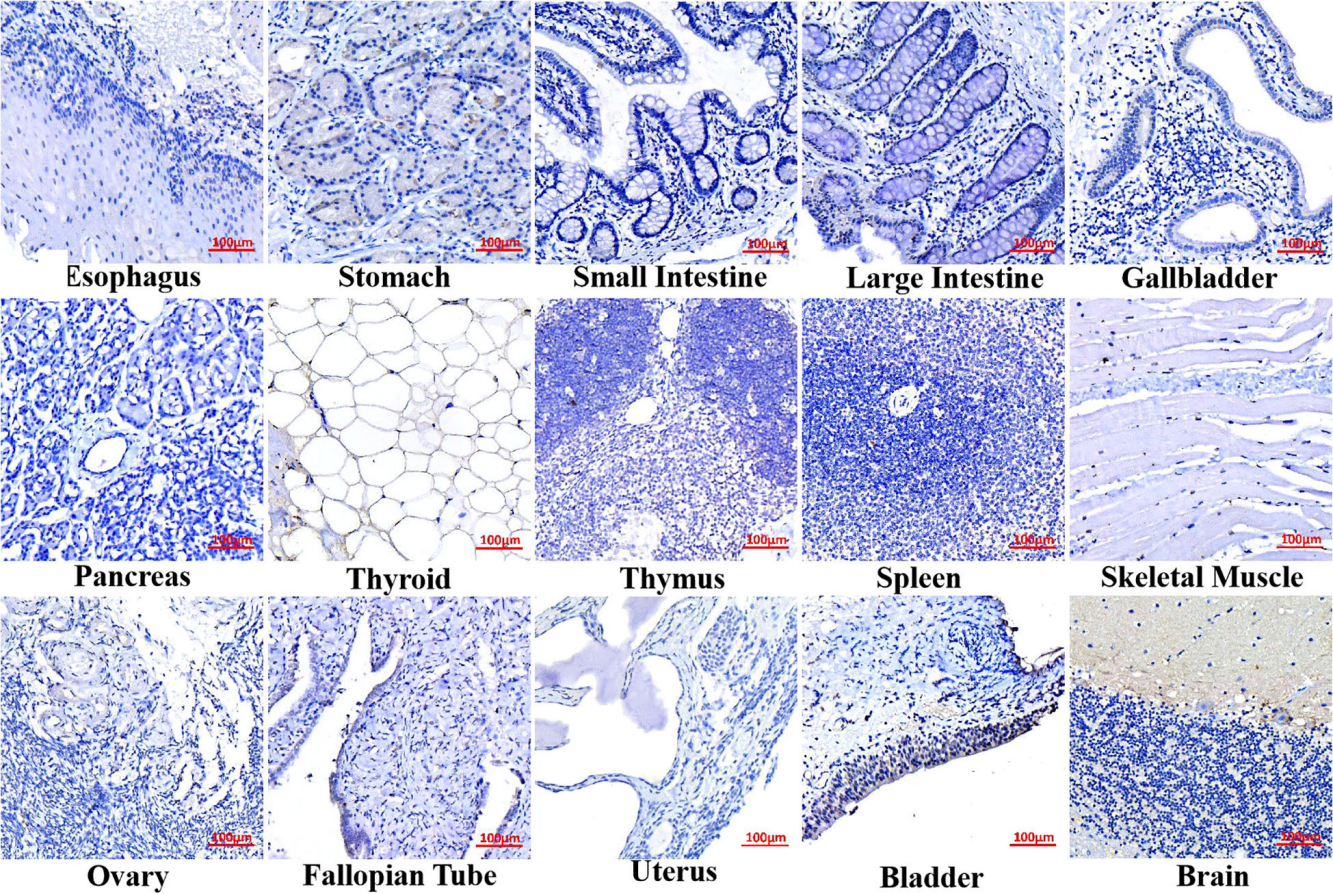
GRM4 expression in human normal tissues. GRM4 distribution was mapped in normal tissues by IHC. Negative GRM4 expression in human esophagus, small intestine, large intestine, gallbladder, pancreas, thyroid, thymus, spleen, skeletal muscle, ovary, fallopian tube and uterus. Positive GRM4 expression in stomach, bladder and brain.

In order to evaluate the expression of GRM4 in normal tissues, we detected it by IHC. The design of a phase I clinical trial incorporates multiple crucial elements. These include the identification of dose-limiting toxicity, determination of target toxicity level, and accurate definition of the maximum tolerated dose. CAR-T therapy may cause on-target off-tumor side-effects, which can be attributed to over-expression of target proteins in normal tissues (9). Based on the HPA, we verified that GRM4 is predominantly expressed in the brain (**Figure 2**). We collected samples from the esophagus, stomach, small intestine, large intestine, gallbladder, pancreas, thyroid, thymus, spleen, skeletal muscle, ovary, fallopian tube, uterus, and bladder. IHC indicated that GRM4 is expressed only in the brain, with minimal expression in the stomach and urothelial cells, and no expression in the other samples.

### GRM4 is expressed on the membrane of breast cancer cell lines

**Figure 3:**
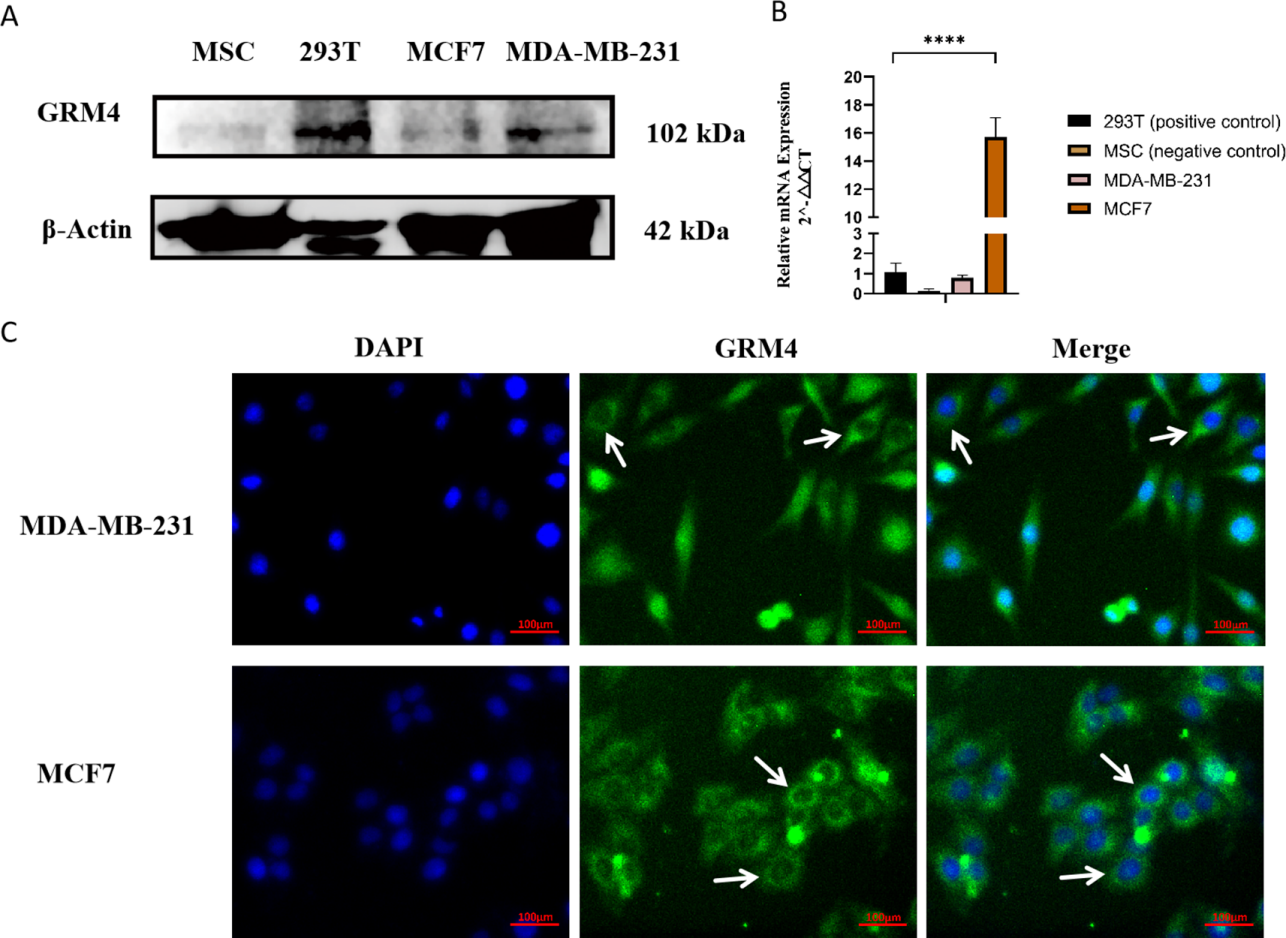
Expression of GRM4 in breast cancer cell lines. GRM4 expression was confirmed in cell lines. **(A)** WB results for GRM4 expression. MCF7 and MDA-MB-231 were positive for GRM4 expression. (293T used for positive control). **(B)** Expression of GRM4 in two BC cell lines (MCF7 and MDA-MB-231), MSC used for negative control) by qPCR. **(C)** Immunofluorescence shows MDA-MB-231 and MCF7 cells have membrane expression of GRM4 stained in green. Nuclei were stained with DAPI (blue). (Scale Bar: 100 µm).

Given our IHC results, the potential adverse effects of GRM4-CAR-T therapy may be limited, rendering GRM4 a worthy candidate for further exploration. Nevertheless, the membrane expression of GRM4 remains to be validated. To ascertain the expression of GRM4 in breast cancer cell lines, western blotting, and qPCR were carried out. The results demonstrated that GRM4 was expressed in the MDA-MB-231 and MCF7 cell lines. The 293T cell line (positive control) also expressed GRM4, whereas MSCs (negative control) exhibited no expression of GRM4 (**Figures 3A, B**) (**Additional File 3**). IF analysis of MCF7 and MDA-MB-231 confirmed the membrane and cytoplasmic expression of GRM4 in these cell lines (**Figure 3C**). Membrane expression of GRM4 in these two cell lines suggests that GRM4 could be a potential target for CAR-associated treatment in BC. However, the expression of GRM4 in BC patient samples requires further investigation, as gene expression in cell lines does not always align with that in tissue samples.

### High expression of GRM4 in BC patients

**Figure 4:**
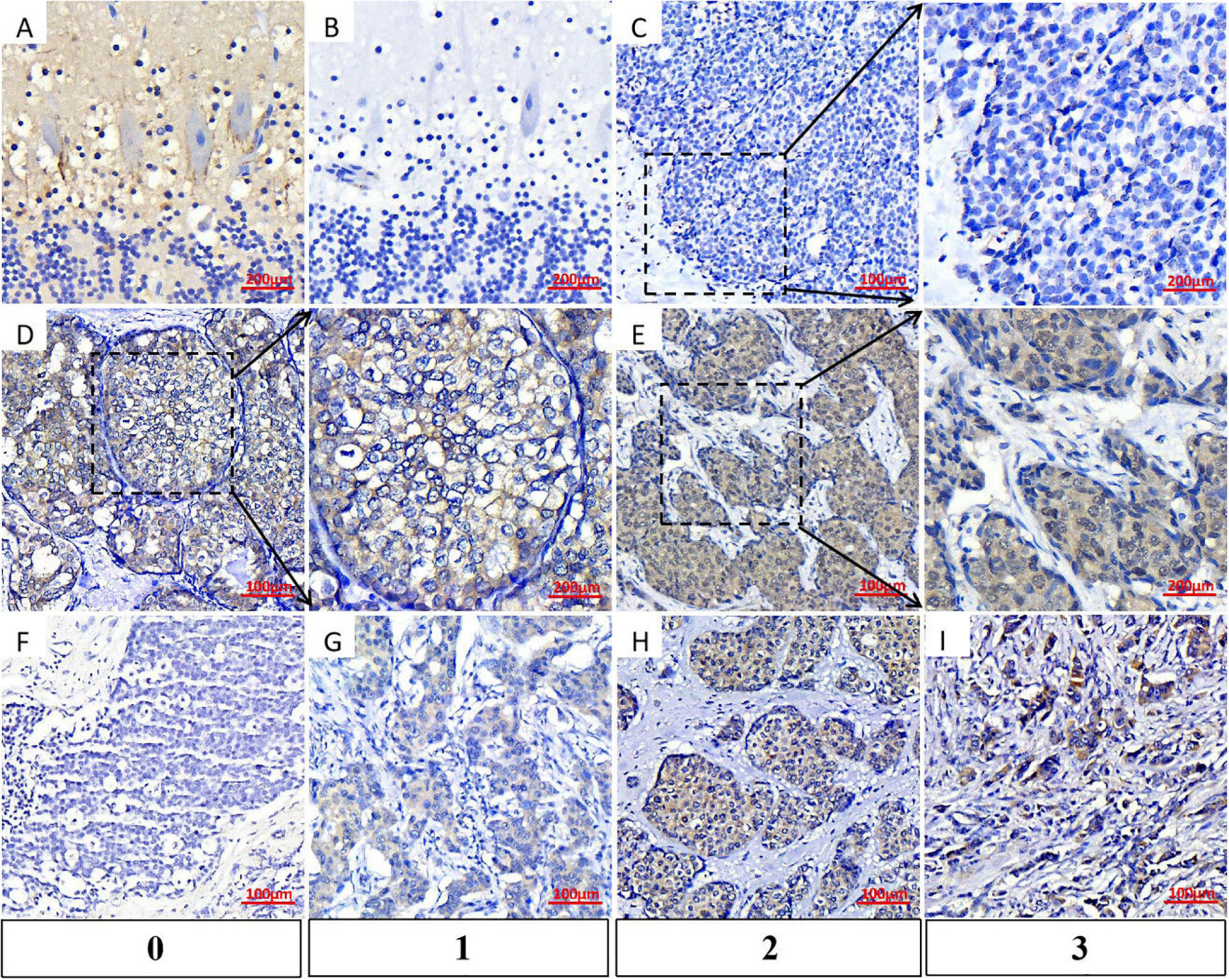
Expression of GRM4 in tissue samples from BC patients. GRM4 expression patterns were characterized in patients.by IHC **(A)** GRM4 expression in cerebellum slices (positive control). **(B)** Cerebellum specimens incubated with PBS were negative (negative control). C-E. Expression of GRM4 in breast cancerous tissues. **(C)** Negative GRM4 expression in BC sample. **(D)** Cell-membrane-positive GRM4 expression in BC sample. **(E)** Cytoplasmic GRM4 expression in BC sample. F-I. Different levels of GRM4 expression were evaluated by H-score. **(F)** Negative GRM4 expression, score 0. **(G)** Weak-positive GRM4 expression, score 1. **(H)** Moderate-positive GRM4 expression, score 2. **(I)** Strong-positive GRM4 expression, score 3.

We performed IHC to investigate the expression of GRM4 in 158 BC patients. Given that GRM4 is regarded as a marker of Purkinje cells in the cerebellum, we employed brain sections abundant in Purkinje cells, which were incubated with anti-GRM4 antibody, as the positive control (**Figure 4A**), and phosphate-buffered saline (PBS) as the negative control (**Figure 4B**). In some BC patients, GRM4 was not expressed (**Figure 4C**). In the magnified image, the expression of GRM4 on the tumor cell membrane was observable (**Figure 4D**). In some patients, GRM4 was expressed in the cytoplasm of tumor cells (**Figure 4E**).

**Figure 5:**
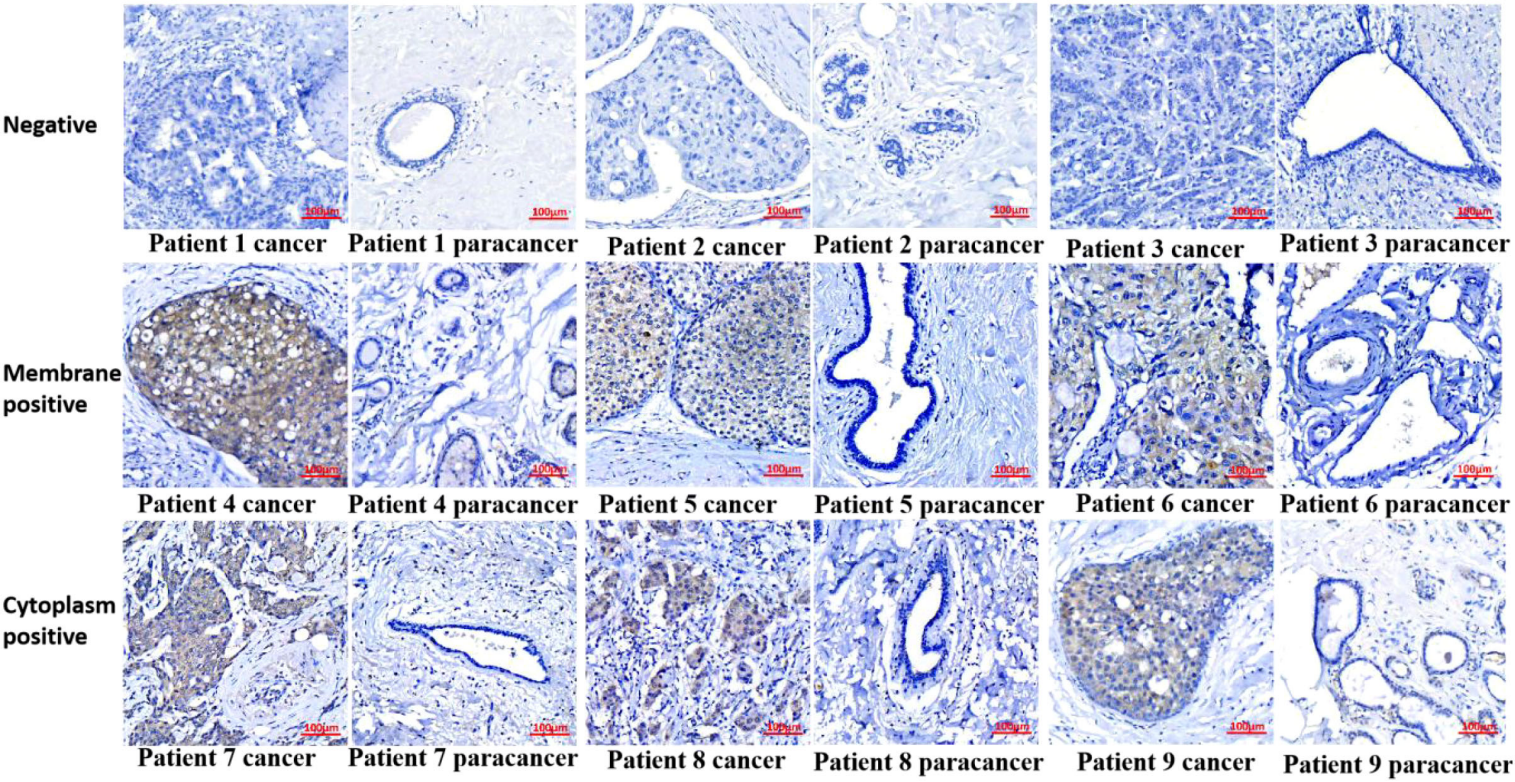
GRM4 expression in breast cancer tissues and para-cancerous tissues from 9 patients. Tumor-specific expression of GRM4 was verified by IHC. Patients 1-3: negative GRM4 expression in cancerous tissue and para-cancerous tissue. Patients 4-6: membrane-positive GRM4 expression in cancerous tissue with negative expression in para-cancerous tissue. Patients 7-9: cytoplasm-positive GRM4 expression in cancerous tissue with negative expression in para-cancerous tissue.

In patients without GRM4 expression in BC tissues, their para-cancerous tissues also did not express this protein (patients 1–3 in **Figure 5**). Notably, in six different patients presented in **Figure 5**, when compared with para-cancerous tissues, GRM4 was expressed solely in the cancerous tissues, regardless of whether it was expressed on the cell membrane (patients 4-6) or in the cytoplasm (patients 7-9). This finding indicates that if GRM4 is utilized as a target, it may not attack normal breast tissue, resulting in minimal or even no side effects on breast tissues (**Figure 5**).

Furthermore, subcellular localization was analyzed. In our patient samples, IHC staining showed that GRM4 was expressed in 80.38% (127/158) of patients, whereas 19.62% (31/158) samples had little or no expression of GRM4 in the tumor cells. In positive patients, 71 cases (44.94%) were both membrane- and cytoplasm-positive, and 56 cases (35.44%) were membrane-positive (**Table 1**). GRM4 is broadly expressed in breast cancer patients. Thus, GRM4 CAR-T therapy may exert a therapeutic effect on almost 80% of patients. Additionally, we found that more than 50% of tumor cells expressed GRM4 in half of the GRM4-positive patients (**Table 2**), and 37.34% of patients were GRM4-positive among which 80% of tumor cells expressed GRM4.

**Table 1:** GRM4 immunohistochemical staining (n=158).

Location of GRM4	n	%
Negative	31	19.62%
Cell membrane and cytoplasmic positive	71	44.94%
Cell membrane positive	56	35.44%

**Table 2:** Tumor proportion score (TPS) of GRM4 (n=158).

TPS Category	n	%
Positive number	127	80.38%
TPS>50%	81	51.27%
TPS>80%	59	37.34%

Our IHC findings were categorized by H-scores, where the staining intensity was defined as follows: 0 denoted an absent expression of the target antigen within tumor cells (**Figure 4F**), 1 signified weak positive expression (**Figure 4G**), 2 corresponded to moderate positivity (**Figure 4H**), and 3 represented a high degree of positive expression (**Figure 4I**).

### GRM4 expression is tumor-specific with no detection in paired para-cancerous tissues

A promising specific target needs high expression in cancerous tissues but no expression in para-cancerous tissues. In our study, we found that in GRM4-positive patients (**Figure 5**), such as patients 4 to 9, GRM4 was expressed in the membrane and cytoplasm of breast cancer cells, while no GRM4 was expressed in normal breast tissues. In GRM4-negative patients, such as patients 1 to 3, GRM4 was not expressed in normal tissues. Collectively, these findings establish GRM4 as a promising diagnostic marker in breast cancer.

### GRM4 expression in different subtypes of breast cancer patients

The expression of GRM4 in five distinct subtypes of breast cancer (**Table 3**) (**Additional File 3**) was characterized. GRM4 was expressed in luminal A, luminal B, triple-negative, Her-2(+)HR(+), and Her-2(+)HR (–) breast cancer, suggesting that GRM4 is broadly expressed in breast cancer. As shown in **Table 1**, GRM4 was expressed in 80.38% of patients. Among Her-2(+)HR(+) patients, 85.29% were GRM4-positive; in Her-2(+)HR(-) patients, 84.85% were GRM4-positive; in triple-negative patients, 75.00% were GRM4-positive; in luminal A, 70.00% of patients were GRM4-positive; and in luminal B, 84.85% of patients were GRM4-positive. GRM4 expression was higher in Her-2-positive patients. In summary, regardless of the breast cancer subtype, GRM4 expression was at least 70% in any subtype, indicating its potential as a promising target for CAR-T therapy.

**Table 3:** GRM4 expression in different types of BC (n=158).

Type	Positive number	Total	Percent
Triple-negative	21	28	75.00%
Her-2+(HR+)	29	34	85.29%
Her-2+(HR-)	28	33	84.85%
Luminal A	21	30	70.00%
Luminal B	28	33	84.85%

### GRM4 expression correlates with advanced stage and older age

We further analyzed the expression of GRM4 and its correlation with clinical parameters (**Table 4**). Interestingly, BC patients with different GRM4 expression levels had statistically significant differences in age (p=0.026) and clinical stage (p=0.025). GRM4 expression was significantly higher in patients over 40 years old and in those with advanced clinical stage. No correlations were found between GRM4 expression and depth of tumor invasion (p=0.193), lymph node metastasis (p=0.162), histological grade (p=0.492), perineural invasion (p=1.000), lymphovascular invasion (p=0.306), histological type (p=0.188).

**Table 4:** GRM4 expression levels and clinical characteristics in BC tissues (n=158).

Clinical parameters	Total	Percent	GRM4 Negative		GRM4 Positive		P-value
	n	%	n	%	n	%	
Total number	158	100.00%	31	19.62%	127	80.38%	
Age							
≤40	10	6.33%	5	50.00%	5	50.00%	
							***0.026***
>40	148	93.67%	26	17.57%	122	82.43%	
Depth of tumor invasion							0.193
pT1	49	31.01%	13	26.53%	36	73.47%	
pT2-pT3	109	68.99%	18	16.51%	91	83.49%	
Lymph node metastasis							0.162
Negative	83	52.53%	20	24.10%	63	75.90%
Positive	75	47.47%	11	14.67%	64	85.33%
Clinical stage							
I	32	20.25%	11	34.38%	21	65.63%	***0.025***
II-III	126	79.75%	20	15.87%	106	84.13%	
Histological grade							
1	9	5.70%	3	33.33%	6	66.67%	
2	46	29.11%	10	21.74%	36	78.26%	0.492
3	97	61.39%	16	16.49%	81	83.51%	
other	5	3.16%	1	20.00%	4	80.00%	
Perineural invasion							1.000
Negative	154	97.47%	31	20.13%	123	79.87%	
Positive	4	2.53%	0	0.00%	4	100.00%	
lymphovascular invasion							0.306
Negative	144	91.14%	30	20.83%	114	79.17%	
Positive	14	8.86%	1	7.14%	13	92.86%	
Histological type							
Invasive ductal carcinoma	136	86.08%	24	17.65%	112	82.35%	
Invasive lobular carcinoma	8	5.06%	2	25.00%	6	75.00%	0.188
other	14	8.86%	5	35.71%	9	64.29%	

Bold values indicate statistical significance (P < 0.05).

### GRM4 expression and correlation with overall survival and disease-free survival in BC

**Figure 6:**
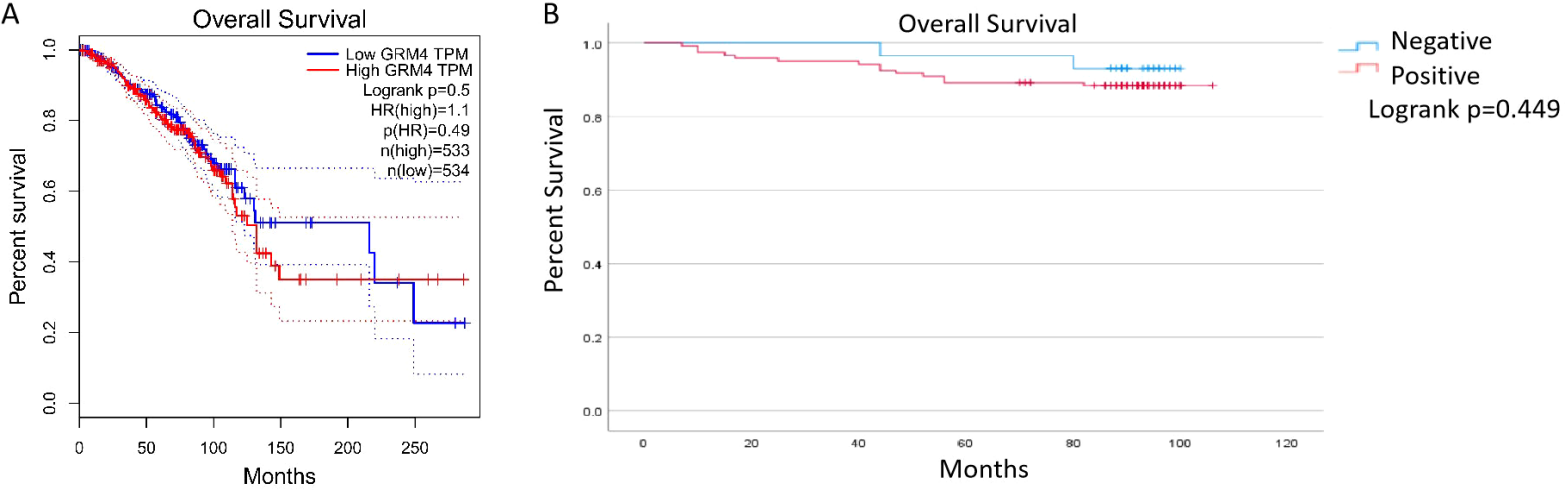
GRM4 expression and survival analysis of breast cancer patients. Survival analysis evaluated GRM4 clinical relevance. **(A)** GRM4 expression and overall survival analysis based on BC clinical data from the GEPIA database (P = 0.5>0.05). **(B)** GRM4 expression and overall survival analysis from our patient cohort (P = 0.449).

Next, we explore the relationship between overall survival (OS) and GRM4 expression. Kaplan–Meier survival curves based on BC patient GRM4 expression in the GEPIA online database were drawn (**Figure 6A**). Additionally, Kaplan–Meier survival curves were made based on 158 patients with BC from the Cancer Hospital of Shantou University Medical College (**Figure 6B**). As shown in **Figure 6A**, GRM4 expression was not related to OS of patients from the GEPIA online database (p=0.5). The GEPIA analysis is based on The Cancer Genome Atlas (TCGA) BRCA dataset, a well-characterized public cohort encompassing the major molecular subtypes and stages of breast cancer. **Figure 6B** shows that the OS of our patient cohort, regardless of GRM4 positivity, was the same as those who were GRM4-negative (p=0.449).

## Discussion

In this study, we report the ectopic expression of GRM4 in breast cancer patients and indicated it is a potential target for CAR-associated therapy. Firstly, GRM4 expression is limited to the brain. Examination of breast cancer cell lines MDA-MB-231 and MCF7 for expression of GRM4, by WB and IF staining, in addition to IHC staining of 158 breast cancer specimens, showed that GRM4 is widely expressed in the cytoplasm and cell membrane of breast cancer cells. GRM4 not only serves as a target for CAR therapies in breast cancer but also can be used in the pathological diagnosis of breast cancer.

GRM4, a member of the GRM protein family, is highly expressed in the central nervous system (CNS). It participates in diverse physiological and pathophysiological processes within the CNS, such as learning, memory, and cognitive impairment (29). Research on the roles of GRM4 in cancers remains scarce and contentious. Although its expression correlates with poor prognosis in colorectal cancer (30) and drug resistance in colon cancer (31), GRM4 agonists can inhibit the proliferation of non-CNS-origin cultured cancer cells, such as bladder cancer cells (32). Kansara et al. recently reported GRM4 regulated myeloid cell tumorigenesis (33). In breast cancer, GRM4 inhibits the proliferation of breast cancer cell lines (34), yet its expression in clinical breast cancer samples remains unclear.

Currently, CAR-T therapy has attained remarkable success in treating hematological malignancies (4). Nevertheless, its application to solid tumors is constrained by the scarcity of specific targets (35). In the context of CAR-T treatment for solid tumors, targets with high coverage or expression on cancer cells are extremely scarce (15). Most of the reported targets for solid tumors exhibit off-target effects, which impede treatment efficacy. Therefore, identifying suitable, highly tumor-specific targets is crucial for minimizing toxicity and ensuring the success of CAR-T therapy. Additionally, in the realm of CAR-T and CAR-NK therapy for breast cancer, several targets, such as EpCAM (NCT02915445), VEGFR (NCT05477927), EGFR, and B7-H3 (NCT05341492), have been explored in clinical trials. However, the number of clinically applicable targets remains limited.

Few researchers have concentrated on identifying ectopically expressed proteins and exploring their roles in cancer. Given the presence of the blood-brain barrier, constructing CAR-NK cells using this target holds potential safety advantages (28). Due to the risk of neurotoxicity from targeting a brain-expressed antigen, GRM4 is better suited for CAR-NK therapy, which has demonstrated a superior safety profile with minimal rates of severe CRS and ICANS in clinical studies (36, 37). While GRM4 could also be considered a candidate for antibody-drug conjugate (ADC) therapy, this strategy would be contingent on efficient antigen internalization. However, the efficacy of ADC using GRM4 need to be explored.

GRM4 is expressed in Purkinje cells and neurons in the brain. In other normal organs, GRM4 shows limited expression in the stomach and bladder. Infusing GRM4 CAR-T cells into patients may cause immune effector cell-associated neurotoxicity syndrome (ICANS). The central mechanism of ICANS—the second most frequent CAR-T cell toxicity (38)—is systemic cytokine release, which disrupts the blood-brain barrier via endothelial activation and promotes neuroinflammation. This BBB dysfunction and resultant cerebral edema are key pathological features (39). Unlike CAR-T cells, which are associated with high levels of IL-6 and other neurotoxicity-linked cytokines, CAR-NK cells exhibit a more favorable cytokine release profile (e.g., dominant in IFN-γ), correlating with a significantly lower incidence of severe neurotoxicity (40). Therefore, if GRM4-CAR-NK cells are to target breast cancer cells, the on-target off-tumor side-effect will be limited. Although some targets such as B7-H3 (https://www.proteinatlas.org/ENSG00000103855-CD276/tissue) expressing in brain, B7-H3 CAR-T cells are widely explored in solid tumor therapy (NCT06158139, NCT06646627, NCT06101082). The expression of an antigen in the brain does not necessarily preclude its potential as a CAR target. Further in-depth exploration is required, and it shows potential therapeutic value for phase I clinical trials.

An ideal CAR-T target should possess three key characteristics: high-level expression on tumor cells, high specificity (manifested solely on tumor cells), and high stability (without any loss of expression) (35). These qualities are fundamental in guaranteeing the efficacy of CAR-T treatment. To be a potential target in CAR-associated therapy, proteins must be specific and broadly expressed in tumor cells. Nowadays, in breast cancer, Her-2 is a marker used clinically. However, it is acknowledged that the Her-2/neu gene undergoes amplification and/or overexpression in 25%-30% of human breast cancer cases (41). In our study, we found that it is expressed in almost 80% of breast cancer which exceeds the percentage of Her-2-expressing tumors. GRM4 is broadly expressed in breast cancer patients, suggesting GRM4 CAR-T therapy may exert a positive effect on almost 80% of patients. Additionally, we found that more than 50% of tumor cells expressed GRM4 in half of BC patients. This means that 51.27% of patients may benefit from GRM4 CAR-T therapy, and this therapy may kill more than 50% of tumor cells. As for about 37.34% of BC patients, it may kill more than 80% of tumor cells. It is a promising target for CAR-associated therapy. However, the efficacy of GRM4 CAR-associated cell therapy is critically dependent on its infiltration into solid tumor sites.

In this study, we found that GRM4 expression is related to patient age. GRM4 is mostly expressed in patients who are over 40 years old. Several studies have revealed distinct molecular characteristics of tumors in relation to age in various cancer types including breast cancer (42, 43). As with Her-2 (44) and ER (45), GRM4 may be a novel biomarker that is related to breast cancer patient age changes.

Our study is the first to report that GRM4 expression was significantly higher in patients over 40 years old, and it may be an age-related gene playing an important role in cancer development. However, its mechanism remains unclear, and the molecular character corresponding to BC patient age needs exploration. Developing drugs against this target or utilizing existing inhibitors could offer a more effective and less toxic treatment option for elderly breast cancer patients. In our study, we found that GRM4 expression is related to the clinical stage, with cancer at an advanced stage, GRM4 expression may increase, so we hypothesize that GRM4 may promote cancer cell development. However, our study patients cohort is mostly at early stage. The expression of GRM4 in later stage in breast cancer need exploration. On the other hand, Bin Xiao (34) reported that GRM4 may inhibit cancer cell proliferation, migration, and invasion. The exact biological role of GRM4 in breast cancer remains unclear and requires further investigation. This association suggests that older patients and those with advanced-stage disease may represent a target population for future GRM4-directed therapies, informing patient selection in clinical trials.

Some studies have illustrated that GRM4 correlates with poor prognosis in colorectal cancer. As for breast cancer (30), GRM4 expression is not related to the OS. This not only is supported by bio-informatic data in GEPIA database from TCGA patient cohort, but also analysis of our own clinical samples, indicating that GRM4 is not a decisive factor in the OS of breast cancer patients. We infer that it may be due to the small number of patients enrolled in our study. In the next step, we will enroll more patients to confirm the results.

In conclusion, we demonstrate that GRM4 is highly expressed in BC. Based on our findings, we anticipate that GRM4 could be a potential CAR-NK target in BC patients for which few therapeutic options exist. However, more large-scale studies and clinical trials will ensure its potential usefulness as a CAR-NK target in BC.

## Data Availability

The original contributions presented in the study are included in the article/**Supplementary Material**. Further inquiries can be directed to the corresponding authors.

## Supplementary material

Additional file 1 Primer sequences.

Additional File 2 Differential gene analysis by three different R packages. Three algorithms were used for differential analysis. **(A)** DESeq2 package showed 90 genes were downregulated while 170 were upregulated in breast cancer tissues compared to normal breast tissues. **(B)** The edgeR package showed 143 genes were downregulated while 242 were upregulated in breast cancer tissues compared to normal breast tissues. C. The limma package showed 138 genes were downregulated while 33 were upregulated in breast cancer tissues compared to normal breast tissues.

Additional File 3 The expression of GRM4 in cell lines by WB. GRM4 is detected in MCF7 and MDA-MB-231 cancer cell lines. MSC is negative control and 293T is positive control.

Additional File 4 GRM4 expression in different types of BC. GRM4 prevalence was assessed across subtypes. GRM4-positive expression in 75% triple-negative patients, 85.29% Her-2+(HR+) patients, 84.85% Her-2+(HR-) patients, 70% Luminal A patients, 84.85% Luminal B patients.
