# Supplementary figures and images for: Glutamate metabotropic receptor 4 in breast cancer: a potential and specific target for chimeric antigen receptor therapy
Source: Front Oncol. 2026 Jan 7;15:1654977. doi: 10.3389/fonc.2025.1654977 (PMC12819273; doi:10.3389/fonc.2025.1654977)

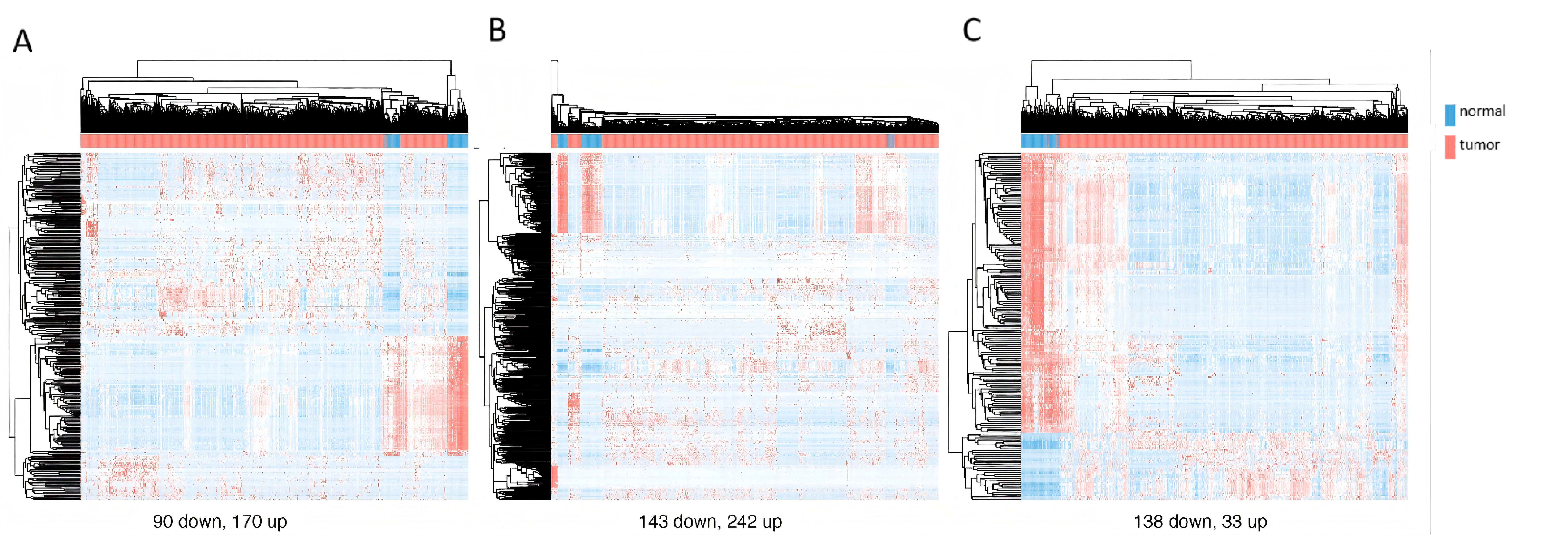

Supplement: Additional File 3 — The expression of GRM4 in cell lines by WB. GRM4 is detected in MCF7 and MDA-MB-231 cancer cell lines. MSC is negative control and 293T is positive control. [file Image1.jpeg]

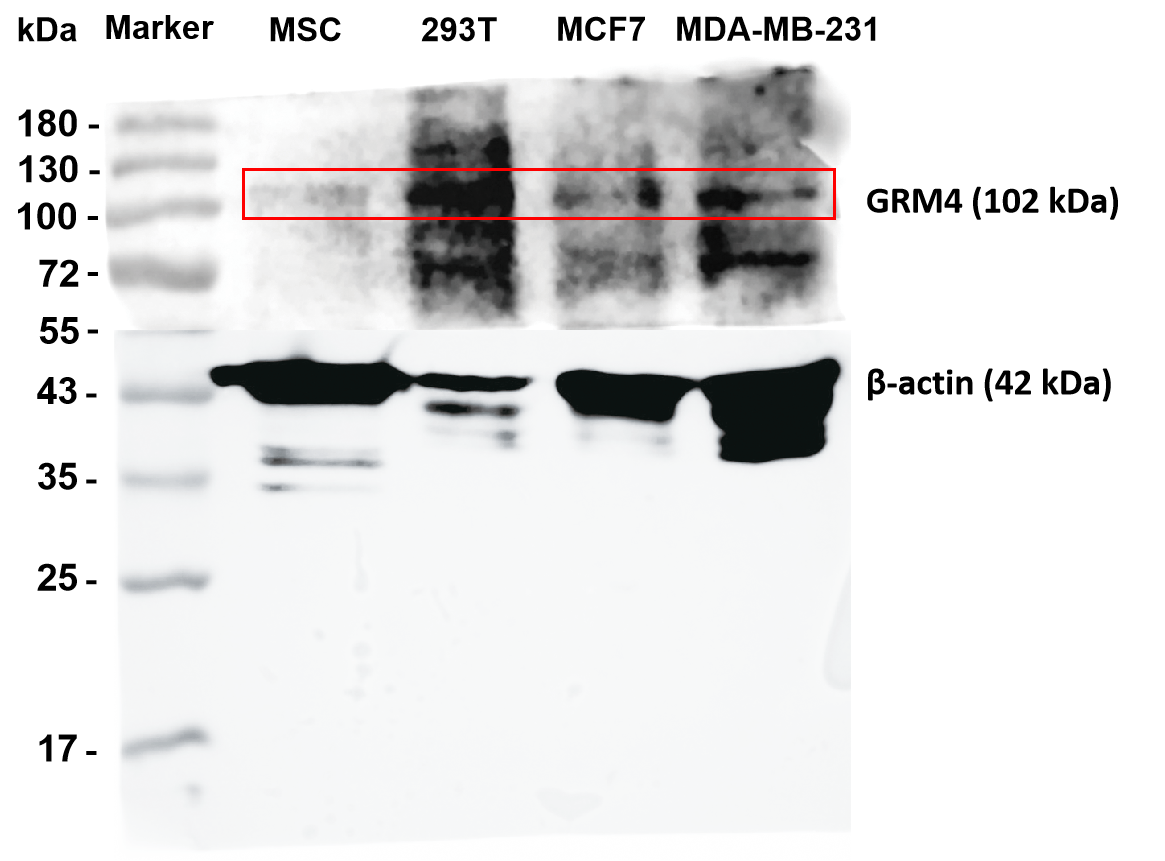

Supplement: Additional File 4 — GRM4 expression in different types of BC. GRM4 prevalence was assessed across subtypes. GRM4-positive expression in 75% triple-negative patients, 85.29% Her-2+(HR+) patients, 84.85% Her-2+(HR-) patients, 70% Luminal A patients, 84.85% Luminal B patients. [file Image2.tif]

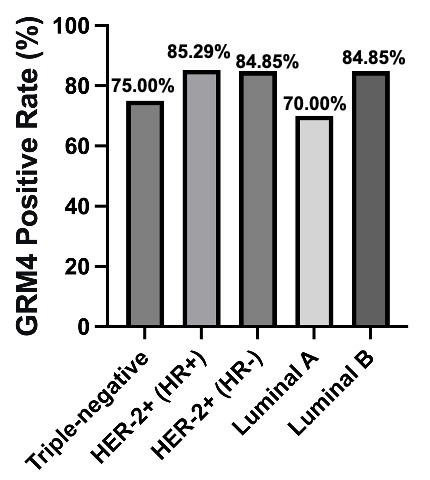

Supplement: Supplementary file 5 [file Image3.jpeg]
